# Genetic markers associated with resistance to beta-lactam and quinolone antimicrobials in non-typhoidal *Salmonella* isolates from humans and animals in central Ethiopia

**DOI:** 10.1186/s13756-017-0171-6

**Published:** 2017-01-17

**Authors:** Tadesse Eguale, Josephine Birungi, Daniel Asrat, Moses N. Njahira, Joyce Njuguna, Wondwossen A. Gebreyes, John S. Gunn, Appolinaire Djikeng, Ephrem Engidawork

**Affiliations:** 1 Aklilu Lemma Institute of Pathobiology, Addis Ababa University, P.O. Box 1176, Addis Ababa, Ethiopia; 2 Biosciences Eastern and Central Africa-International Livestock Research Institute (BecA-ILRI) Hub, P O Box 30709, Nairobi, Kenya; 3 Department of Microbiology, Immunology & Parasitology, School of Medicine, College of Health Sciences, Addis Ababa University, Churchill Avenue, P.O. Box 9086, Addis Ababa, Ethiopia; 4 ICIPE-African Insect Science for Food and Health, P.O. Box 30772-00100, Nairobi, Kenya; 5 Department of Veterinary Preventive Medicine, The Ohio State University, 1920 Coffey Rd., Columbus, OH 43210 USA; 6 Department of Microbial Infection and Immunity, Center for Microbial Interface Biology, The Ohio State University, Biomedical Research Tower, 460 West 12th, Columbus, OH 432101214 USA; 7 Department of Pharmacology and Clinical Pharmacy, School of Pharmacy, College of Health Sciences, Addis Ababa University, Churchill Avenue, P.O. Box 1176, Addis Ababa, Ethiopia

**Keywords:** Non-typhoidal *Salmonella*, Antimicrobial resistance, Mechanisms of resistance, Beta-lactamase, Quinolone, Fluoroquinolone, Human strains, Animal strains, Ethiopia

## Abstract

**Background:**

Beta-lactam and quinolone antimicrobials are commonly used for treatment of infections caused by non-typhoidal *Salmonella* (NTS) and other pathogens. Resistance to these classes of antimicrobials has increased significantly in the recent years. However, little is known on the genetic basis of resistance to these drugs in *Salmonella* isolates from Ethiopia.

**Methods:**

*Salmonella* isolates with reduced susceptibility to beta-lactams (*n* = 43) were tested for genes encoding for beta-lactamase enzymes, and those resistant to quinolones (*n* = 29) for mutations in the quinolone resistance determining region (QRDR) as well as plasmid mediated quinolone resistance (PMQR) genes using PCR and sequencing.

**Results:**

Beta-lactamase genes (*bla*) were detected in 34 (79.1%) of the isolates. The dominant *bla* gene was *bla*TEM, recovered from 33 (76.7%) of the isolates, majority being TEM-1 (24, 72.7%) followed by TEM-57, (10, 30.3%). The *bla*OXA-10 and *bla*CTX-M-15 were detected only in a single *S.* Concord human isolate. Double substitutions in *gyr*A (Ser83-Phe + Asp87-Gly) as well as *par*C (Thr57-Ser + Ser80-Ile) subunits of the quinolone resistance determining region (QRDR) were detected in all *S.* Kentucky isolates with high level resistance to both nalidixic acid and ciprofloxacin. Single amino acid substitutions, Ser83-Phe (*n* = 4) and Ser83-Tyr (*n* = 1) were also detected in the *gyr*A gene. An isolate of *S*. Miami susceptible to nalidixic acid but intermediately resistant to ciprofloxacin had Thr57-Ser and an additional novel mutation (Tyr83-Phe) in the *par*C gene. Plasmid mediated quinolone resistance (PMQR) genes investigated were not detected in any of the isolates. In some isolates with decreased susceptibility to ciprofloxacin and/or nalidixic acid, no mutations in QRDR or PMQR genes were detected. Over half of the quinolone resistant isolates in the current study 17 (58.6%) were also resistant to at least one of the beta-lactam antimicrobials.

**Conclusion:**

Acquisition of *bla*TEM was the principal beta-lactamase resistance mechanism and mutations within QRDR of *gyr*A and *par*C were the primary mechanism for resistance to quinolones. Further study on extended spectrum beta-lactamase and quinolone resistance mechanisms in other gram negative pathogens is recommended.

## Background

Salmonellosis in humans is caused by several serovars belonging to *Salmonella enterica* subspecies enterica. Infection by *Salmonella* causes two forms of diseases; typhoid fever, a febrile illness caused by a few host specific serovars such as *Salmonella enterica* subspecies enterica serovar Typhi (*S.* Typhi,) and *S*. Paratyphi A, while the majority of *Salmonella* serovars cause non-typhoidal salmonellosis characterized by self limiting gastoentritis and occasional invasive salmonellosis in immunocompromised, young and elderly patients. Infection with non-typhoidal *Salmonella* (NTS) serovars is one of the leading causes of foodborne illnesses worldwide [1]. NTS infection is commonly associated with consumption of contaminated food of animal origin such as poultry products, beef and pork as well as contact with infected animals [2–4].

Antimicrobial treatment is usually not recommended due to the self-limiting nature of the disease. However, in cases of invasive complicated salmonellosis, treatment with beta-lactam antimicrobials such as ampicillin, ceftriaxone and quinolone drugs are employed as lifesaving agents [5]. Resistance to beta-lactam antimicrobials and quinolones has increased dramatically in NTS isolates from humans as well as food animals worldwide [6–9]. The common mechanism of resistance to beta-lactam antimicrobials is due to production of beta-lactamase enzymes with variable level of activity against different generations of beta-lactam antimicrobials. In addition to the first generation beta-lactamases: *bla*TEM1, *bla*SHV1, several extended spectrum *bla*TEM and *bla*SHV variants, other extended spectrum beta-lactamase enzymes such as *bla*CTX-M, *bla*CMY, *bla*OXA and AmpC have been reported in *Salmonella* serotypes from different parts of the world [10–13].

Resistance to quinolone drugs is primarily mediated by mutations in Quinolone Resistance Determining Region (QRDR) of *gyr*A and *par*C genes in *Salmonella* and other Gram-negative organisms. Specifically, high level resistance to ciprofloxacin is frequently attributed to double mutations in the *gyr*A gene and single or double mutation in the *par*C gene [14]. In addition to chromosomal mutations, other mechanisms such as activation of efflux pumps (multidrug efflux pump and quinolone specific plasmid mediated efflux pump encoded by *qep* genes), *qnr* (plasmid-mediated quinolone resistance), porins, and quinolone-modifying enzyme (*aac(6')-Ib-cr*) have been associated with decreased susceptibility to quinolones [14]. Of particular concern is the occurrence, within the last few years in different parts of the world, of plasmid-mediated quinolone resistance encoded by several *qnr* genes. These genes encode for pentapeptide proteins that protect bacterial topoisomerases from the effect of quinolones. They do not induce high level resistance but their presence leads to mutation in the QRDR [15]. However, recent report from Senegal indicated the presence of *qnrB1* together with the quinolone modifying enzyme *aac(6’)-Ib-cr* in *Salmonella* associated with high level resistance to ciprofloxacin even in the absence of mutations in the QRDR [16]. These resistance determinants have been observed in various gram negative organisms including *Salmonella* [16, 17]. In recent years, the rate of resistance to ciprofloxacin has increased considerably in both clinical and food isolates of *Salmonella* [6, 18, 19].

In Ethiopia, reports revealed resistance to beta-lactam antimicrobials and quinolones in *Salmonella* isolates from human patients and food of animal origin [20, 21]. However, little data is available on the genetic basis of the observed phenotypic drug resistance. Multidrug resistant *S.* Concord isolates obtained from children adopted from Ethiopia in different European countries and USA were reported to harbor *bla*CTX-M-15, *bla*TEM1, *bla*SHV-12 genes encoding for resistance to third generation cephalosporins, *qnrA* and *qnrB* encoding for reduced susceptibility to fluoroquinolones [22, 23]. The aim of this study was to investigate the genetic markers associated with resistance to beta-lactam and quinolone antimicrobials among NTS isolates collected from humans and animals in central Ethiopia.

## Methods

### Bacterial isolates

Non-typhoidal *Salmonella* strains investigated in the current study were isolated from feces of food animals (cattle *n* = 50, poultry *n* = 26, swine *n* = 8) in Addis Ababa and surrounding districts of Oromia region namely: Ada, Barake, Sebeta and Sululta. In addition, *Salmonella* isolates obtained from stool of temporally and spatially related diarrheic human patients from primary health centers and Tikur Anbessa Specialized Hospital in Addis Ababa (*n* = 68) were also included. All human and animal isolates were collected from 2013 to 2014.

### Antimicrobial susceptibility testing, serotyping and phage typing

Susceptibility of each isolate to beta-lactam and quinolone antimicrobials was determined using disk diffusion method according to the guidelines of the Clinical and Laboratory Standards Institute (CLSI). The interpretation of the categories of susceptible, intermediate or resistant was also based on the CLSI guidelines [24]. For purposes of analysis, all readings classified as intermediate were considered resistant unless otherwise mentioned. *Escherichia coli* ATCC 25922 was used as a quality control. *Salmonella* isolates were serotyped and phage-typed at the Public Health Agency of Canada, World Organization for Animal Health (OIÉ), Reference Laboratory for Salmonellosis, Guelph, Ontario, Canada as described previously [25].

### Bacterial DNA extraction

Isolates were grown on Luria Bertani (﻿LB) agar (37 °C, over night). A single colony was inoculated to 5 ml of LB broth and grown in a shaking incubator at 37 °C for 16–18 h. Genomic DNA was then extracted using the QIAGEN genomic DNA extraction kit (QIAGEN, USA) according to the manufacturer’s recommendation.

### Detection and characterization of beta-lactamase enzymes

A total of 43 isolates, 12 from humans and 31 from animals, with reduced susceptibility to one or more of beta-lactam antimicrobials (ampicillin, amoxicillin + clavulanic acid, cephalothin, ceftriaxone) were tested for genes encoding for beta-lactamase enzymes. PCR and DNA sequencing were performed for the detection and characterization of beta-lactamase (*bla*) genes with oligonucleotide primers previously described for *bla*TEM, *bla*SHV, *bla*PER, *bla*PSE, *bla*OXA1*, bla*OXA4*, bla*OXA10*, bla*CMY*,* and *bla*CTX-M genes (Table 1). The PCR conditions for all reactions involved an initial denaturation for 3 min at 95 °C followed by 30 cycles of (95 °C for 30 s, specific annealing temperature for 1 min, and extension at 72 °C for 30 s) followed by a final extension at 72 °C for 5 min. Specific annealing temperature for each PCR reaction is shown in Table 1. Group specific primers were used to characterize *bla*CTX-M enzymes [26]. The PCR amplicons were purified using QIAGEN PCR purification kit (QIAGEN, USA) and sequenced with forward and reverse primers at Sequencing, Genotyping, Oligosynthesis and Proteomics (Segolip) unit of Biosciences eastern and central Africa (BecA). All amplicon sequences were assembled and translated to amino acid sequences using CLC Main Work Bench (Inqaba Biotechnical Industries, (Pty) Ltd, Denmark) and compared with protein sequences in the Genbank database. Classification of *bla*TEM enzymes was based on beta-lactamase classification database (https://www.lahey.org/studies/temtable.asp).

**Table 1:** List of primers used for detection and characterization of beta-lactamases

Gene/target	Primer	Sequence 5’-3’	Amplicon size	AT °C	Ref	Remark
BLA_Tem_ Gene	TEM-F1	ATGAGTATTCAACATTTCCG	862-bp	55	[34]	
	TEM-R1	GACAGTTACCAATGCTTAATCA				
	blaTEM-F2	TAA CCA TGAGTGATAACACT			[34]	sequencing
	blaTEM-R2	CCGATCGTT GTCAGAAGTAA				
BLA _SHV_ gene	Bla SHV-F1	CTTTACTCGCCTTTATCG	827-bp	56	[34]	
	Bla SHV-R1	TCCCGC AGATAAATCACCA				
	blaSHV-F2	ACTGCCTTTTTG CGCCAGAT			[34]	sequencing
	blaSHV-R2	CAGTTCCGTTTCCCAGCGGT				
Bla OXA-1	OXA-1-F	ATGAAAAACACAATACATATCAAC	755-bp	48	[13]	
	OXA-1-R	TTTCCTGTAAGTGCGGACAC				
Bla OXA −4	OXA-4-F	TCAACAGATATCTCTACTGGT	216 bp	54	[13]	
	OXA-4-R	TTTATCCCATTTGAATATG				
Bla OXA-10	OXa 10-F	TCAACAAATCGCCAGAGAAG	277 bp	57	[13]	
	Oxa-10-R	TCCCACACCAGAAAAACCA				
bla PER	Per1-F	AATTTGGGCTTAGGGCAGAA	925 bp	55	[50]	
	Per1-R	ATGAATGTCATTATAAAAGC				
blaPSE	blaPSE-F	TGCTTCGCAACTATGACTAC			[42]	
	blaPSE-R	AGCCTGTGTTTGAGCTAGAT				
blaCYM	blaCMY2-F	TGGCCGTTGCCGTTATCTAC	868	57	[9]	
	blaCMY2-R	CCCGTTTATGCACCCATGA				
CTX-M group I	CTXM1-F3	GACGATGTCACTGGCTGAGC	499	55	[38]	
	CTXM1-R2	AGCCGCCGACGCTAATACA				
CTX-M group II	TOHO1-2 F	GCGACCTGGTTAACTACAATCC	351	55	[38]	
	TOHO1-1R	CGGTAGTATTGCCCTTAAGCC				
CTX-M group III	CTXM825F	CGCTTT GCCATGTGCAGCACC	307	55	[38]	
	CTXM825R	GCT CAGTACGATCGAGCC				
CTX-M group IV	CTXM914F	GCTGGAGAAAAGCAGCGGAG	474	62	[38]	
	CTXM914R	GTAAGCTGACGCAACGTCTG				

### Investigation of quinolone resistance mechanism

Isolates with reduced susceptibility to nalidixic acid and/or ciprofloxacin (*n* = 29), three human isolates and 26 animal isolates were examined for the presence of known quinolone resistance determinants. Quinolone resistance determining region (QRDR): *gyr*A*, gyr*B*, par*C and *par*E genes were amplified using PCR. PCR was also used to examine for various plasmid mediated quinolone resistance genes: *qnr*A*, qnr*B*, qnr*D*, qnr*S*, qep*A*,* and *aac(6′)-Ib-cr* as described previously (Table 2). Similar PCR conditions described previously were used and annealing temperature for each primer set is presented in Table 2. PCR ampilicons were purified using QIAGEN PCR purification kit and sequenced as previously described. Presence of mutation in the QRDR was examined by translating nucleotide sequences into proteins and aligning against reference sequence of *S.* Typhimurium strain LT2 on NCBI database (Accession Number AE006468).

**Table 2:** List of primers used for detection of quinolone resistance mechanism

Gene	Primer name	Primer sequence (5′ to 3′)	Product size	AT in °C	References
*gyr*A	GyrAFP	AAATCTGCCCGTGTCGTTGGT	344 bp	58	[16]
	GyrARP	GCCATACCTACTGCGATACC			
*gyr*B	GyrB FP	GAATACCTGCTGGAAAACCCAT	446 bp	57	[16]
	GyrB RP	CGGATGTGCGAGCCGTCGACGTCCGC			
*par*C	ParC FP	AAGCCGGTACAGCGCCGCATC	395 bp	57	[16]
	ParC RP	GTGGTGCCGTTCAGCAGG			
*Par*E	ParE FP	TCTCTTCCGATGAAGTGCTG	240 bp	55	[12]
	ParE RP	ATACGGTATAGCGGCGGTAG			
*qnr*A	qnrA FP	ATTTCTCACGCCAGGATTTG	516 bp	53	[43]
	qnrA RP	GATCGGCAAAGGTTAGGTCA			
*qnr*B	qnrB FP	GATCGTGAAAGCCAGAAAGG	469 bp	53	[43]
	qnrB RP	ACGATGCCTGGTAGTTGTCC			
*aac*(6’)-Ib	aac(6’)-Ib FP	TTGCGATGCTCTATGAGTGGCTA	482-bp	55	[36]
	*aac(6’)-Ib-RP*	CTCGAATGCCTGGCGTGTTT			
	*aac(6’)-Ib-cr-seq*	CGTCACTCCATACATTGCAA (for sequencing of aac(6’)-Ib-cr			
*qep*A	QepA FP	CGTGTTGCTGGAGTTCTTC	403 bp	59	[7]
	QepA RP	CTGCAGGTACTGCGTCATG			
*Qnr*D	QnrD FP	CGAGATCAATTTACGGGGAATA	565 bp	53	[8]
	QnrD RP	AACAAGCTGAAGCGCCTG			
*Qnr*S	QnrS FP	ACGACATTCGTCAACTGCAA	417 bp	53	[43]
	QnrS RP	TAAATTGGCACCCTGTAGGC			

*FP* forward primer, *RP* Reverse primer

## Results

### Resistance to beta-lactam antimicrobials and beta-lactamase genes in *Salmonella* isolates from animals and humans

Of the 20 different serotypes investigated in the current study, resistance to at least one beta-lactam antimicrobial was detected in nine serotypes and the *bla* gene was detected only in isolates belonging to six serotypes. Of the 43 isolates resistant to one or more beta-lactam antimicrobials (ampicillin, amoxicillin + clavulanic acid, cephalothin, ceftriaxone), *bla* genes were detected in 34/43 (79.1%) of the isolates. The dominant *bla* gene responsible for resistance to beta-lactam antimicrobials in the majority of *Salmonella* isolates, 33 (76.7%) was found to be variants of *bla*TEM gene. Most of these were TEM-1 type, 24 (72.7%) followed by TEM-57, 10 (30.3%). Both phenotypic resistance to beta-lactam antimicrobials and detection of *bla* genes was more common in isolates obtained from poultry compared to isolates from other sources (Table 3). In one of the human isolates of *S.* Concord, two *bla* genes (*bla*OXA-10 and *bla*CTX-M-15*)* were detected. Both of these genes encode for enzymes capable of extended spectrum beta-lactamase activity. This isolate was resistant to the third generation cephalosporin, ceftriaxone in addition to ampicillin, and cephalothin. In eight (18.6%) of the isolates, none of the tested *bla* genes were detected (Table 3).

**Table 3:** Occurrence of *bla* genes in *Salmonella* isolates from different sources with reduced susceptibility to beta-lactam antimicrobials

Source	Total no. of isolates	^a^Resistant to ≥ one of beta-lactams (%)	*bla* genes detected^b^			Not detected	No.(%) positive for bla genes
			*blaTEM*	*blaOXA10*	*blaCTX-M*		
Cattle	50	16(32)	12	-	-	4	8(75)
Poultry	26	13(50)	12	-		1	12(92.3)
Swine	8	2(25)	1	-	-	1	1(50)
Human	68	12(17.7)	9	1	1	2	^c^10(83.3)
Total	152	43(28.3)	34	1	1	8	34(79.1)

^a^Ampicillin, Cephalothin, Cefoxitin, Ceftriaxone, amoxicillin and clavulanic acid

^b^Though all isolates were screened for *bla*SHV, *bla*OXA1,* bla*OXA4, *bla*PER, *bla*PSE and *bla*CMY2, none of them were positive for these genes

^c^ *blaOXA10* and *blaCTX-M-15* were detected in a single isolate

Among the dominant serotypes, 66.7, 92.3, 50 and 100% of *S.* Typhimurium, *S*. Saintpaul, *S*. Virchow and *S*. Kentucky were positive for variants of the *bla*TEM gene, respectively. All of the 10 *S.* Kentucky isolates collected from cattle, poultry and humans were resistant to ampicillin, cephalothin and amoxicillin + clauvlanic acid and were all positive for *bla*TEM-1 gene (Table 4).

**Table 4:** Beta-lactam resistance profile among different *Salmonella* serotypes from various hosts, occurrence of betalactamses genes and *bla*-types based on amino acid sequences

Serotype	Total	Host (No.)	No. resistant to Beta-lactam(%)	Beta-lactam R-profile		*bla* positive(%)	*bla* type
				Intermediate	Resistant		
Aberdeen	1	C	-	-	-	-	-
Agona	1	C	-	-	-	-	-
Braenderup	3	C(2), H(1)	-	-	-	-	-
Concord	1	H	1(100)	Fox	AmpAmcCroCf	1(100)	OXA-10 and blaCTX-M-15
Dublin	3	C	2(66.7)	Cf	-	-	-
Enteritidis	2	H	-	-	-	-	-
Haifa	4	C(3), P(1)	1P(25)	Amp	-	-	-
Heidelberg	1	S	1(100)	Amc	AmpCf	1(100)	TEM-1
I:6;7,14:-:I,w	1	C	1(100)	-	AmpAmcCf	1(100)	TEM-1
I: Rough-O:I:1,2	1	S	-	-	-	-	-
Kentucky	10	C(6),	6(100)	-	AmpAmcCf	6(100)	TEM-1
		P(2)	2(100)	-	AmpAmcCf	2(100)	TEM-1
		H(2)	2(100)	Fox	AmpAmcCf	2(100)	TEM-1
				Amc	AmpCf		
Kottbus	8	C(1), H(7)	-	-	-	-	-
Livingstone var.14+	2	C(1), S(1)	-	-	-	-	-
Miami	5	H(3), S(2)	-	-	-	-	-
Mikawasima	2	C(2)	-	-	-	-	
Newport	2	H(2)	-	-	-	-	-
Saintpaul	33	P(20)	10(50)	AmcCf	Amp	4(100)	TEM-57
				-	AmpAmcCf	4(100)	TEM-57
				Amc	AmpCf	2(100)	TEM-57
		C(10)	2(20)	Amc	AmpCf	2(100)	TEM-1
		S(2)	-	-	-	-	-
		H(1)	1(100)	Amc	AmpCf	1	TEM-1
Typhimurium	42	C(12)	2(16.7)	-	AmpAmcCf	2(100)	TEM-1
		P(3)	-	-	-	-	-
		S(1)	1(100)				
		H(26)	6(64.5)			5(83.3)	TEM-1
				Cf	-	-	-
				Amc	AmpCf	2(100)	TEM-1
				Amp	-	1(100)	TEM-1
				-	AmpAmcCf	2(100)	TEM-1
Virchow	28	C(6)	3(50)	Amc	AmpCf	1(33.3)	TEM-1
		H(22)	1(4.6)	-	AmpAmcCf	1(100)	TEM-1
V:ROUGH-O;-;-	1	H(1)	1	-	AmpCf	-	-

*C* Cattle, *H* Human, *P* poultry, *S* swine, − = not detected, *Amp* ampicillin, *Amc* amoxicillin and clavulanic acid, *Cf* cephalothin, *Cro* ceftriaxone

Interestingly, all 10 *bla*TEM-57 were recovered from *S.* Saintpaul isolated from poultry, while those *S.* Saintpaul strains obtained from cattle and human were all TEM-1 type. Despite a change in amino-acid sequences, there was no distinct difference in phenotypic antimicrobial susceptibility pattern to beta-lactam antimicrobials among isolates carrying *bla*TEM-57 and *bla*TEM-1 enzymes.

Among eight isolates in which none of the tested *bla* genes were detected, most of them were susceptible to the major beta-lactams and were at the margin of susceptibility and intermediate; *S.* Dublin (*n* = 2) and *S.* Typhimurium (*n* = 2) only to cephalothin and *S.* Haifa to ampicillin (*n* = 1) On the other hand, three *S.* Virchow isolates and one *S.* V:ROUGH-O;-:- were completely resistant to ampicillin and cephalothin.

### Mechanism of resistance to quinolone antimicrobials

Out of the 29 *Salmonella* isolates with reduced sensitivity to quinolones, high level resistance to both nalidixic acid and ciprofloxacin was observed in only 10 *S.* Kentucky isolates (34.5%) (Table 5). All of these *S.* Kentucky isolates had double mutations in *gyr*A (Ser83-Phe + Asp87-Gly) and *par*C (Thr57-Ser + Ser80-Ile) genes. Single mutation in *gyr*A (Ser83-Phe) was observed in four isolates (*S.* Livingstone var.14+ (2), *S.* Virchow (1), *S*. I:6;7,14:-:I,w (1). All these isolates were resistant to nalidixic acid and intermediately resistant to ciprofloxacin. A single amino acid substitution in *gyr*A (Ser83-Tyr) was detected in one *S.* Haifa from poultry with an R-phenotype [resistant to nalidixic acid and intermediately resistant to ciprofloxacin]. Overall, double and single substitutions in *gyr*A were detected in 15 (51.7%) of the isolates. Double substitution in *par*C (Thr57-Ser + Tyr83-Phe) was detected in one *S.* Miami isolated from swine. This strain was sensitive to nalidixic acid and intermediately resistant to ciprofloxacin. The Tyr83-Phe is a novel mutation. A strain of *S.* Agona with only single substitution at Thr57-Ser was intermediately resistant to nalidixic acid but sensitive to ciprofloxacin. Double substitution in the *gyr*B gene (Val423-Gly + Asp459-His) was detected in two isolates; *S*. Mikawasima and *S.* Braenderup, the latter having additional substitution in *par*C gene (Thr57-Ser) associated with intermediate susceptibility to both nalidixic acid and ciprofloxacin, whereas the former with intermediate susceptibility only to naldixic acid. A strain of serotype V: rough-O;-:- that was susceptible to nalidixic acid but intermediately resistant to ciprofloxacin had single substitution of Ser463-Ala on *gyr*B gene. Over half of the quinolone resistant isolates in the current study 17 (58.6%) were also resistant to at least one of the beta-lactam antimicrobials (Table 5) and all *S.* Kentucky isolates resistant to nalidixic acid and ciprofloxacin were also shown to be MDR to several antimicrobials in our previous works [27, 28]. No mutation was detected in *par*E gene in any of the isolates examined in the current study. Nine isolates with reduced sensitivity to nalidixic acid and or ciprofloxacin had no mutation in any of the QRDR (Table 5).

**Table 5:** Susceptibility of isolates to quinolone drugs and mutation in QRDR

	Serotype	Zone of inhibition mm (susceptibility category)		R-pattern betalactams^a^	Mutation in QRDR		
		Na	Cip		*gyrA*	*gyrB*	*parC*
Cattle	Aberdeen	19[S]	27[I]	-	-	-	-
Cattle	Virchow	20[S]	25[I]	*-*	-	-	-
Cattle	Typhimurium PT 3	21[S]	25[I]	**AmpAmcCf**	-	-	-
Cattle	Typhimurium PT 4	22[S]	27[I]	**AmpAmcCf**	-	-	-
Cattle	Haifa	21[S]	25[I]	*-*	-	-	-
Poultry	Saintpaul	20[S]	31[S]	*AmcCf*	-	-	-
Cattle	Saintpaul	17[I]	25[I]	**AmpAmcCCf**	-	-	-
Cattle	Saintpaul	21[S]	27[I]	-	-	-	-
Poultry	Saintpaul	20[S]	27[I]	*-*	-	-	-
Human	V:ROUGH-O;-:-	22[S]	28[I]	**AmpCf**	-	Ser463Ala	-
Cattle	Mikawasima	20[S]	25[I]	-	-	Val423Gly + Asp459His	-
Cattle	Agona	17[I]	31[S]	-	-	-	Thr57Ser
Cattle	Braenderup	17[I]	25[I]	-	-	Val423Gly + Asp459His	Thr57Ser
Swine	Miami	22[S]	27[I]	-	-	-	Thr57Ser + Tyr83Phe
Poultry	Haifa	0[R]	25[I]	-	Ser83Tyr	-	-
Cattle	Virchow	0[R]	26(I)	**Amp**Amc**Cf**	Ser83Phe	-	-
Cattle	Livingstone var.14+	0[R]	24(I)	-	Ser83Phe	-	-
Cattle	I:6;7,14:-:I,w	7[R]	30(I)	**AmpAmcCf**	Ser83Phe	-	-
Swine	Livingstone var.14+	0[R]	20[I]	*-*	Ser83Phe	-	-
Cattle	Kentucky	0[R]	14[R]	**AmpAmcCf**	Ser83Phe + Asp87Gly	-	Thr57Ser + Ser80Ile
Cattle	Kentucky	0[R]	12[R]	**AmpCf** *Amc*	Ser83Phe + Asp87Gly	-	Thr57Ser + Ser80Ile
Cattle	Kentucky	0[R]	11[R]	**AmpCf** *Amc*	Ser83Phe + Asp87Gly	-	Thr57Ser + Ser80Ile
Cattle	Kentucky	0[R]	9[R]	**AmpAmcCf**	Ser83Phe + Asp87Gly	-	Thr57Ser + Ser80Ile
Cattle	Kentucky	0[R]	12[R]	**AmpCf** *Amc*	Ser83Phe + Asp87Gly	-	Thr57Ser + Ser80Ile
Cattle	Kentucky	0[R]	10[R]	**AmpAmcCf**	Ser83Phe + Asp87Gly	-	Thr57Ser + Ser80Ile
Human	Kentucky	0[R]	8[R]	**AmpAmcCf** *Fox*	Ser83Phe + Asp87Gly	-	Thr57Ser + Ser80Ile
Human	Kentucky	0[R]	10[R]	**AmpCf** *Amc*	Ser83Phe + Asp87Gly	-	Thr57Ser + Ser80Ile
Poultry	Kentucky	0[R]	9[R]	**AmpAmcCf**	Ser83Phe + Asp87Gly	-	Thr57Ser + Ser80Ile
Poultry	Kentucky	0[R]	11[R]	**AmpAmcCf**	Ser83Phe + Asp87Gly	-	Thr57Ser + Ser80Ile

*PT* Phagetype, *Amp* Ampicillin, *Amc* Amoxicillin and clavulanic acid, Cf Cephalothin, *Cip* Ciprofloxacin, *Na-* Nalidixic acid, *S* susceptible, *I* intermediately resistant, *R* resistant

^a^Resistance status, isolates were fully resistant to antimicrobials written in Bold and intermediately resistant to those written in italics

### Plasmid mediated quinolone resistance

None of the tested plasmid mediated quinolone resistance genes were detected in the isolates examined in the current study. Seven isolates belonging to serotypes Saintpaul, Typhimurium, Aberdeen, Virchow and Haifa were susceptible to nalidixic acid but had shown reduced sensitivity to ciprofloxacin according to CLSI (2013) cut-off points, with zone of inhibition ranging from 25 to 28 mm. There appears to be other resistance mechanisms responsible for the observed decreased sensitivity.

## Discussion

Detection of a high resistance rate to beta-lactam antimicrobials (50%) and the presence of *bla* genes in isolates from poultry could be due to the fact that drugs like ampicillin and amoxicillin are frequently employed in poultry farms in Ethiopia leading to selection pressure. The dominant beta-lactamase genes detected in the current study were variants of *bla*TEM with the majority being *bla*TEM-1, which is concordant with the observed spectrum of resistance to only ampicillin and first generation cephalosporin in most of the isolates. In Africa, *bla*TEM-1 has been reported from *Salmonella* isolated from poultry in Egypt [29], from children adopted from Mali [30], and *S.* Enteritidies in Senegal [31].

All *S.* Saintpaul isolated from poultry in the current study carried *bla*TEM-57, while those from cattle and human carried *bla*TEM-1. This is probably due to mutation of a *bla*TEM gene in a strain of *S.* Saintpaul in one of the poultry farms and clonal spread of strain carrying this mutant gene to farms in the area. All poultry *S.* Saintpaul were isolated from farms in the Adaa district. Compared to TEM-1, TEM-57 has a substitution of Gly to Asp at position 92 of amino acid sequence, which was first reported from *Proteus mirabilis* [32] and later on from *E. coli* in China [33]. To our knowledge, this is the first report of detection of *bla*TEM-57 in *Salmonella*. Fortunately, this mutation was not associated with extended spectrum activity against second and third generation cephalosporins.

One of the human isolates, *S.* Concord, resistant to ampicillin, cephalothin, cefoxitin and ceftriaxone was shown to produce *bla*CTX-M-15 and *bla*OXA-10. Previous studies have also reported *bla*CTX-M-15 in *S.* Concord isolated from children adopted from Ethiopia to different European countries and USA [34, 35]. In fact, a separate study also showed that *bla*CTX-M-15 isolated from *S.* Concord from Ethiopia was chromosomally encoded [35]. Nevertheless, the previous studies also showed production of *bla*SHV-12 in most of the *S.* Concord from Ethiopia, but OXA-10 was not reported. This is presumably due to loss of a plasmid encoding for SHV-12 and acquisition of OXA-10 in a new isolate from Ethiopia. During the last few years, CTX-M-15 and other related CTX-M enzymes have been widely reported from various *Enterobacteriaceae* including *Salmonella* in different African countries from both hospital and community settings [16, 36–39]. Oxacilinases including OXA-10 have also been commonly isolated from different enterobacteriaceae including *Salmonella* [40].

The possible reason for the absence of *bla* genes in a few isolates with reduced susceptibility in the current study despite testing for most of the known *bla* reported in *Salmonella* could be due to poor sensitivity of phenotypic resistance detection methods. In two *S*. Typhimurium and two *S*. Dublin intermediately resistant only to cephalothin and one *S.* Haifa intermediately resistant to ampicillin, the reading was at the margin of intermediate and susceptible. However, all the three *S*. Virchow were fully resistant to ampicillin and cephalothin and intermediately resistant to amoxicillin + clauvlanic acid. For these isolates, other resistance mechanisms not investigated in this study such as alterations in the beta-lactam targets (PBPs) [41], absence or down-regulation of the production of outer membrane porins [42], over expression of efflux pumps [43] and different *amp*C betalactamases [44] might be responsible for the observed reduction in susceptibility. In general, the rate of occurrence of extended spectrum beta-lactamases in *Salmonella* isolates in the current study is low. This could be due to the fact that most of the human isolates were obtained from primary health care centers and use of 2nd and 3rd generation cephalosporins is not a common practice in veterinary medicine [28, 45]. The single MDR *S.* Concord in the current study was isolated from hospitalized 1 year old child.

Amino acid substitutions at codon 83 and 87 of *gyr*A gene have been associated with high level fluoroquinolone resistance [46–49] whereas resistance to only nalidixic acid is associated with single or double mutation in *par*C gene in *Salmonella* and other Gram-negative pathogens [14, 50]. Detection of two amino acid substitutions in the *gyr*A gene at codon 83 and 87 and the *par*C gene at codon 57 and 80 in all *S.* Kentucky isolates with high level resistance to both nalidixic acid and ciprofloxacin obtained from humans and animals suggests the possibility of clonal spread of *S.* Kentucky strain in the human and animal population in the study area. Similar mutations in *gyr*A and *par*C genes were reported from *S.* Kentucky from French travelers returning from north east and eastern Africa [51]. Studies of *S.* Kentucky ST198 from different countries have also shown a similar substitution in *gyr*A at codon 83 (Ser83-Phe) for all isolates and substitution of aspartate at codon 87 with asparagine, tyrosine or glycine residues. *S.* Kentucky isolates in the current study also belonged to ST198 suggesting the clonal relatedness of our isolates to the internationally spreading clone of *S.* Kentucky (unpublished data). However, only single substitution in the *par*C gene at codon 80 (Ser80-Ile) was reported previously and none of them had substitution at codon 57 of *par*C gene [52]. Additional substitution at codon 57 of the *par*C gene in the Ethiopian isolates might have occurred separately. Contrary to these local and global spread of MDR fluoroquinolone resistant *S.* Kentucky, a previous study on *S.* Typhimurium showed that mutation based fluoroquinolone resistance is associated with fitness cost and resistant strains are less invasive [53]. This suggests that this internationally dispersed clone of *S.* Kentucky has unique mechanisms. Furthermore, we have previously shown that *S.* Kentucky strains from Ethiopia has strong biofilm forming ability which is one of the important traits for persistence of the organism in the host or the environment [54] that might have contributed to its dissemination.

Four of the *Salmonella* isolates resistant to nalidixic acid and intermediately resistant to ciprofloxacin had only a single substitution in the *gyr*A, Ser83-Phe, whereas one isolate *S*. Haifa from poultry had a Ser83-Tyr substitution. Previous studies have also shown that a single mutation in *gyr*A results only in resistance to nalidixic acid and not to ciprofloxacin [47, 53]. Although isolates with a single mutation in *par*C gene resulted only with reduced susceptibility to nalidixic acid, an *S.* Miami isolate with no mutation in *gyr*A gene but double substitution in *par*C gene: (Thr57-Ser) and a novel substitution (Tyr83-Phe) was fully susceptible to nalidixic acid and intermediately resistant to ciprofloxacin. This suggests that the novel mutation at codon 83 of *par*C gene might accentuate the activity of nalidixic acid and attenuate the activity of ciprofloxacin.

The observation of double substitution in *gyrB* gene (Val423-Gly + Asp459-His) associated with intermediate susceptibility only to nalidixic acid shows a minor contribution of mutation in *gyr*B compared to *gyr*A for development of resistance﻿ to quinolones. Interestingly, nine isolates with reduced sensitivity to ciprofloxacin and some to nalidixic acid had no mutation in QRDR. We have also not detected PMQR genes in any of the isolates. Other resistance mechanisms not tested in this study such as multidrug efflux pumps, other PMQR mechanisms recently described in *Salmonella* such as oqxAB efflux pump [19], and altered outer membrane porins might be involved [14].

## Conclusion

Co-occurrence of beta-lactamases with ciprofloxacin resistant determinants in large proportion of isolates is a major threat. Occurrence of MDR *S.* Kentucky with high level fluoroquinolone resistance mediated by double mutations in *gyr*A and *par*C genes in cattle, poultry, and human in the study area suggests clonal spread of this strain and the need for strict pathogen control strategies to hamper further spread of this pathogen. As the majority of the isolates in this study were from healthy animals at the farm level and human patients from primary health care centers, the data presented here may not represent the national status. Further studies on extended spectrum beta-lactamase and fluoroquinolone resistance mechanisms in *Salmonella* and other gram negative pathogens in hospital and community settings is recommended.

## Abbreviations

*bla*: Betalactamase gene
CLSI: Clinical and Laboratory Standards Institute
MDR: Multi-drug resistance
NTS: Non-typhoidal *Salmonella*
PMQR: Plasmid mediated quinolone resistance
QRDR: Quinolone resistance determining region
